# Migration of an Inferior Vena Cava Tumor Thrombus during Renal Cell Carcinoma Resection

**DOI:** 10.1155/2023/6632030

**Published:** 2023-12-26

**Authors:** Roupen Hatzakorzian, Andrea Blotsky, Albert Moore, Julien Vaillancourt, Pattra Mettasittigorn, Armen Aprikian, Steven B. Backman

**Affiliations:** ^1^Department of Anesthesia, McGill University Health Centre, Glen Site, Royal Victoria Hospital, 1001 Boulevard Décarie, Montréal H4A 3J1, Québec, Canada; ^2^Department of Critical Care, McGill University Health Centre, Glen Site, Royal Victoria Hospital, 1001 Boulevard Décarie, Montréal H4A 3J1, Québec, Canada; ^3^Department of General Internal Medicine, McGill University, St-Mary's Hospital, 3830 Lacombe Avenue, Montréal H3T 1M5, Québec, Canada; ^4^Department of Urology, McGill University Health Centre, Glen Site, Royal Victoria Hospital, 1001 Boulevard Décarie, Montréal H4A 3J1, Québec, Canada

## Abstract

Approximately 4%–10% of patients with renal cell carcinoma (RCC) have tumoral vascular invasion with resultant thrombi in the renal vein and in the inferior vena cava (IVC). The authors describe an interesting case of IVC tumor thrombus that migrated to the right cardiac chambers during RCC resection. The diagnosis was made by intraoperative transesophageal echocardiography (TEE), which revealed the presence of a free-floating thrombus between the right atrium (RA) and right ventricle (RV). The patient required an urgent sternotomy with cardiopulmonary bypass (CPB) for atrial thrombus removal prior to the completion of the nephrectomy. The patient made a full recovery and was discharged to a rehabilitation facility. These findings illustrate the importance of intraoperative TEE monitoring during nephrectomy and IVC thrombectomy. In this case, TEE allowed for the diagnosis of an unexpected complication necessitating prompt cardiac surgical management.

## 1. Introduction

A tumor thrombus is commonly defined as tumor extension into a blood vessel. Renal cell carcinoma (RCC) infrequently occurs in association with tumor thrombus, and extension of tumor thrombi into the inferior vena cava (IVC) is an even rarer phenomenon, occurring in 4%–10% of patients with RCC [1]. RCC treatment options include surgery, radiation therapy, chemotherapy, biologic therapy, and targeted therapy. Given the poor renal tumoral response to adjuvant therapy protocols, surgical tumor removal remains the cornerstone of RCC treatment [2]. Radical nephrectomy with tumor thrombectomy is the current standard surgical procedure for these tumors, with a demonstrated 5‐year survival rate of up to 60% in RCC patients presenting without metastasis [3, 4]. The surgical treatment of RCC with tumor thrombus poses a unique challenge, as it frequently requires a multidisciplinary surgical approach adapted to the level of vascular involvement of the tumor thrombus. We report a case of an IVC tumor thrombus that migrated to the right cardiac chambers during RCC resection. Intraoperative transesophageal echocardiography (TEE) demonstrated the presence of a free-floating thrombus between the right atrium (RA) and right ventricle (RV).

## 2. Case Presentation

**Figure 1:**
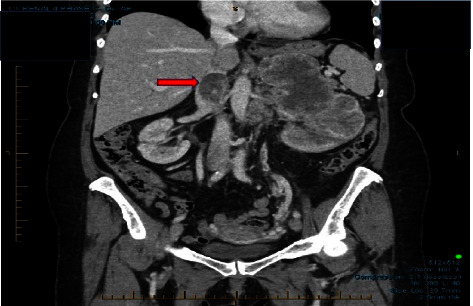
The computed tomography scan coronal images demonstrate tumor thrombus involving the left renal vein extending into the junction of the hepatic/infrahepatic IVC. Arrow indicates tumor thrombus at the junction of the hepatic/infrahepatic IVC.

Consent for publication was obtained from the patient. Following a 4-month history of flank pain and hematuria, a 69-year-old woman was diagnosed with a left-sided RCC tumor and IVC thrombus. She was admitted to hospital for a left nephrectomy and an IVC thrombectomy. The patient's past medical history was positive only for hypertension. Her preoperative medication included amlodipine 5 mg daily. Routine blood work, coagulation profile, and electrocardiogram (ECG) were within normal limits. A computed tomography (CT) scan performed preoperatively demonstrated a large heterogeneous left renal mass (9.1 × 8.6 × 14.3 cm) consistent with an RCC. There was also a large tumor thrombus in the left renal vein extending into the junction of the hepatic/infrahepatic IVC (Figure 1). No tumor thrombus was observed to extend above the hepatic veins or diaphragm.

In the operating room, a #16 gauge *i.v.* was inserted into an upper extremity vein and a thoracic epidural at T8-T9 was placed without difficulty for perioperative pain control. Canadian Anesthesiologists' Society-standard monitors were applied (ECG, noninvasive blood pressure, pulse oximetry, Train of Four). Following preoxygenation, induction of general anesthesia (fentanyl and propofol), muscle paralysis (rocuronium), and oral intubation (#7.0 endotracheal tube) proceeded uneventfully. Invasive monitors including radial artery (left) and central venous catheters (ultrasound-guided right internal jugular vein) were placed and general anesthesia was maintained with sevoflurane. Normothermia was maintained with an upper-body warmed-air blanket.

**Figure 2:**
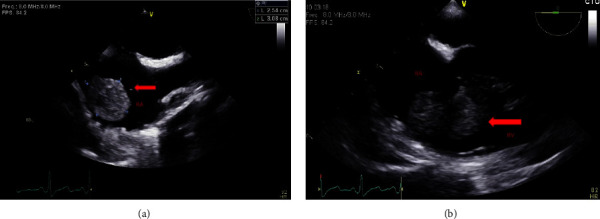
(a) Right atrial tumor thrombus. Midesophageal bicaval view at 110°. RA, right atrium. Arrow indicates right atrial tumor thrombus. (b) Right atrial and ventricular mobile tumor thrombus. Midesophageal 4-chamber right heart view at 0°. RA, right atrium; RV, right ventricle. Arrow indicates mobile tumor thrombus.

Via a chevron incision, the surgical team proceeded to isolate the left kidney and identified the IVC thrombus extending into the junction of the hepatic IVC. It was noted that there was an injury to a small branch on the right lateral aspect of the IVC that required a distal IVC and right renal vein clamp to be placed prior to repair. Upon clamp removal, the patient remained hemodynamically stable with no change in end-tidal CO2; however, the site of the IVC where the tumor thrombus was previously located could no longer be palpated by the surgical team. A TEE probe was inserted promptly, and imaging revealed a 2.5 × 3 cm free-floating thrombus between the RA and the RV (Figures 2(a) and 2(b), VIDEO 1).

After urgent intraoperative consultation with the cardiac surgery team, it was assessed that a sternotomy would be required for the removal of the mass in light of its high risk of embolization. The nephrectomy was thus aborted, and a temporary closure of the left hemichevron incision was performed. A median sternotomy was then executed by the cardiac surgeon. After cannulation of the superior vena cava, IVC, and aorta, the patient was connected to the cardiopulmonary bypass (CPB) circuit. A right atriotomy was executed without aortic cross-clamping and cardioplegia, and the tumor thrombus was extracted without complications. The IVC and RV were inspected; no residual tumor was identified by the cardiac surgeon. The atriotomy was then closed and the patient was weaned off CPB after a total of 10 minutes. The sternum was then closed, and samples were sent to pathology for analysis. The primary surgical team then proceeded with the initial planned intervention; a left nephrectomy was then completed without difficulty.

Throughout both procedures, the patient remained hemodynamically stable with minimal vasopressor requirements. Estimated blood loss was 1.5 L and the patient received a total of 3 L of crystalloid, 4 units of packed red blood cells, and 2 units of fresh frozen plasma. The patient was then transferred intubated in stable condition to the intensive care unit (ICU) and was extubated within 12 hours of ICU admission. The thoracic epidural was used for pain control and was removed on postoperative day 4. She had an uneventful recovery in the ICU and was subsequently discharged to her community hospital for ongoing rehabilitation after a total length of hospital stay of 12 days. Pathology performed on the atrial mass demonstrated an irregular thrombus measuring 2.7 × 3.0 × 1.3 cm with a possible tumor within.

## 3. Discussion

The established treatment strategy for RCC with tumor thrombus is radical nephrectomy and thrombectomy, with the aim of prolonging survival. Although the TNM classification method is the standard staging system in oncology to evaluate the anatomical extent of solid tumors, other systems such as the Neves and Novick system have been proposed to better characterize the extent of local RCC tumor invasion and involvement of the IVC. Both classification systems consider the level of IVC thrombus in relation to the renal veins and the diaphragm. Hepatic vein involvement and the presence of atrial thrombi are also utilized in the Neves and Novick classification, thus allowing the clinician to more precisely discern the extent of IVC involvement. Tumor thrombus in RCC is classified according to the level of the thrombus: extension into renal vein (level I), infrahepatic IVC extension (level II), retrohepatic IVC extension (level III), and extension into RA (level IV) [5, 6]. Regardless of the classification system utilized, high-grade tumors are therefore associated with more proximal involvement of the IVC thrombus.

Our case is unique in that the tumor thrombus migrated intraoperatively to a position between the RA and RV. Whereas other cases of cavoatrial tumor thrombi have been reported, the majority of thrombi were identified preoperatively to extend into the right atrium [7, 8]. The literature suggests that RCC tumor thrombus migration to the RA and beyond is an exceedingly rare event, occurring only in 1% of cases [9, 10]. Our case is unique in so far that the tumor thrombus migrated intraoperatively from the IVC to the RA, ultimately traversing the tricuspid valve and invading the RV. It is important to note that the migration of the tumor thrombus occurred after the surgical manipulation of the IVC. A clamp was placed on the distal IVC to repair an injury to a small vessel prior to tumor resection. Surgical management of RCC tumor with IVC involvement is challenging. This case demonstrates that surgical manipulation of the IVC can cause the dislodgment of an IVC tumor thrombus.

The importance of TEE in the management of RCC with level IV tumors with atrial involvement is well established. TEE is also imperative in cases that require the use of CPB. The indications for TEE monitoring in RCC resections remain unclear. The Practice Guidelines for Perioperative Transesophageal Echocardiography recommends perioperative TEE in noncardiac surgeries “when the patient has known or suspected cardiovascular pathology that might result in hemodynamic, pulmonary, or neurologic compromise” [11]. Calderone et al. recommend the use of preoperative TEE in all cases with a known tumor thrombus with discretion as to what extent TEE is used throughout the remainder of the case [12].

Unfortunately, preoperative imaging obtained within days prior to RCC tumor resection does not necessarily reflect the actual extent of the thrombus at the time of surgery. Consequently, intraoperative TEE can provide the most up-to-date information of the thrombus level. Knowing the precise location and extension of a tumor thrombus is essential for optimal surgical planning. Depending on the tumor thrombus anatomy, a multidisciplinary team including vascular surgeons, cardiac surgeons, cardiac anesthesiologists, and perfusionists may need to be involved [13].

In this case, the patient was hemodynamically stable throughout the case despite the dislodgment of the tumor thrombus. There were no immediate hemodynamic clues to indicate tumor thrombus migration, so the anesthesiologist had no objective evidence alerting to this complication. If the surgeon had not noted the absence of the tumor thrombus in the IVC, a TEE would not have been performed, and the migration would have been missed. This raises the concern for undetected tumor thrombus migration into the RA that could result in cardiovascular collapse. There is a potential risk of intraoperative migration of an IVC tumor thrombus from the level of hepatic and infrahepatic veins into the RA and RV, and dislodgment of the tumor at any IVC level could ultimately result in tumor thrombus positioned in the RA. The risk of migration is increased if there is a surgical manipulation of the IVC prior to tumor resection, as demonstrated in our case. Clamping and unclamping the IVC can affect tumor thrombus migration. Tumor thrombus dislodgment can also occur during IVC thrombectomy. As the cardiovascular vital signs associated with cavoatrial tumor thrombus migration may go unrecognized, it is reasonable to recommend that intraoperative TEE monitoring be utilized in resections of RCC with IVC involvement at any level to track thrombus transit and to ultimately improve patient safety. Our case illustrates that the signs and symptoms of intraoperative thrombus dislodgment may be subtle and definitive treatment may be delayed if TEE monitoring is not implemented early on. We believe that a postinduction, preincision placement of a TEE probe is ideal as it can provide useful and expeditious real-time information regarding intraoperative thrombus location and migration.

Once thrombus migration was ascertained, an interdisciplinary decision was made to interrupt the nephrectomy and proceed directly to atrial thrombectomy that included sternotomy and CPB with attendant anticoagulation implications. The advantage of this staged surgical approach was to address the situation as quickly as possible to minimize the potentially devastating consequences of embolization. The disadvantages of this approach include the interruption of surgical progress for the primary procedure (nephrectomy) and having to resume the procedure afterwards under less-than-ideal hemostasis due to the preceding CPB run. Vinzant et al. recently reported the perioperative outcomes for radical nephrectomy and level III-IV IVC tumor thrombectomy. Patients with level IV tumor thrombus undergoing resection had a greater need for CPB, a higher blood loss, and a longer hospital stay and were more likely to have intraoperative TEE monitoring [14]. Preoperatively, our patient was diagnosed with an RCC and a level II tumor thrombus, but intraoperatively, this changed to a level IV tumor thrombus due to its migration to the right atrium. CPB and open cardiac surgery were necessary to extract the tumor thrombi to avoid a potentially fatal pulmonary embolism. The patient also had an epidural placed for postoperative pain control. Epidural analgesia in surgical interventions that include CPB is controversial due to a theoretical increased risk of epidural haematoma related to systemic anticoagulation [15]. In this case, the anesthetic management included an epidural for intra- and postoperative analgesia patients as the use of CPB and systemic heparinization was not planned. In patients where the RCC includes thrombus invading the IVC, epidural analgesia should be carefully considered as there is always a possibility that CPB and anticoagulation will be required.

## 4. Conclusion

This report demonstrates the importance of intraoperative TEE monitoring during renal nephrectomy and IVC thrombectomy regardless of the level of the IVC tumor thrombi. TEE can monitor tumor thrombi, diagnose tumor emboli, and help guide surgical techniques. The case also highlights that any surgical manipulation of the IVC prior to or during tumor resection can cause tumor thrombus migration.

## Supplementary Materials

Supplementary Materials Right atrial and ventricular mobile tumor thrombus. Midesophageal 4-chamber right heart view at 0°.
